# To what extent residual alveolar ridge can be preserved by implant? A systematic review

**DOI:** 10.1186/s40729-016-0057-z

**Published:** 2016-11-23

**Authors:** Ahmed Khalifa Khalifa, Masahiro Wada, Kazunori Ikebe, Yoshinobu Maeda

**Affiliations:** 1 Department of Prosthodontics, Faculty of Dentistry, Mansoura University, 68 ElGomhoria Street, ElMansoura, 35516 Egypt; 2 Department of Prosthodontics, Gerodontology and Oral Rehabilitation, Osaka University Graduate School of Dentistry, 1-8 Yamadaoka, Suita Osaka, 565-0871 Japan

**Keywords:** Dental implants, Ridge preserving, Alveolar bone remodeling

## Abstract

**Background:**

It has been reported that the load for (or to) implant-supported restoration may lead to bone remodeling as bone resorption and/or formation. While many authors supported the process of bone resorption, others elaborated bone apposition and increasing bone density close and remote to implant body (or fixture). This may suggest the role of the implant to reserve alveolar ridge from physiologic/pathologic resorption. The aim of this systematic review was to predict to how extend dental implants can preserve the residual alveolar ridge based on previous clinical investigations.

**Methods:**

This systematic review based on the retrospective and prospective studies, randomized clinical trial, and case reports. The process of searching for proposed articles included PubMed, Ovid, and Web of Science databases, with specific inclusion and exclusion criterion.

**Results:**

A total 2139 citations were identified. After expunging the repeated articles between databases and application of exclusion and inclusion criteria, 18 articles were found to meet the topic of this systematic review. Many of the articles reported bone preservation with implant-assisted restorations, and the rest denoted noticeable bone apposition.

**Conclusion:**

According to the published clinical studies, the behavior of bone remodeling around implant predicts a sort of residual alveolar bone preservation.

## Review

### Introduction

Edentulism is rated between 7 and 69% internationally [1]. Many biological and non-bilogical predisposing factors lead to the main result of edentulism [2]. Regardless the debate to understand the way of resorption [3], the loss of periodontal ligament by tooth extraction leaves alveolar bone without a chance of reformation which leads to bone resorption only. The resorption shows variation in rate with recorded fast bone loss at the first 6 months after extraction and the following 2 years [4].

As pernicious sequelae of edentulism, the patient lacks most of the ordinary oral function which requires planned rehabilitation. Implant therapy is one of the recent trends to restore oral functions [5–7]. Besides the rehabilitation purposes, implants show other favorable biological effects on the bone state. Many authors [8–10] revealed the ability of the implant to regain bone density at healing and adapt to the applied load. As an evitable fate, residual alveolar ridge shows resorption under the conventional complete denture. This varies according to prosthetic planning, construction, and maintenance, as well as systemic predisposing factors [11–13]. Although the presence of implant beneath complete or partial denture improves denture foundation and augments patient satisfaction [7, 14], there is a controversy about the role of implant overdenture in the process of ridge reshaping after loading.

The tracing of the bone resorption is difficult for the complete denture with the continuous rated atrophy of the residual alveolar ridge beneath the conventional denture [15, 16]. Rather than monitoring the bone atrophy, this review investigates the capability of the implant to be responsible for preserving residual alveolar ridge bone and the role of implant-assisted restoration to reduce the alveolar ridge atrophy.

### Methods

#### Focus question

The (PIO) question to be focused was “In patient with implant restoration, what is the chance of residual alveolar ridge preserving and bone formation in the adaptive remodeling and what are the features of this preservation?”

#### Search strategy

The required documents were collected from PubMed, Web of Science, and Ovid databases. For expanding the traces of researching, further readings for the bibliography of the relevant publications and hand searching for some denoted articles were done. The keywords, for intervention and outcome, used in research engines in databases as “implant overdenture,” “implant bone resorption,” “alveolar ridge preservation,” “improve alveolar ridge,” “implant bone remodeling,” “implant bone reformation,” and “implant bone growth” (Table 1). In all databases, the filters of human, English article, and dental journals were applied.

**Table 1:** Systematic search strategy

Focus question	In patient with implant restoration, what is the chance of residual alveolar ridge preserving and bone formation in the adaptive remodeling and what are the features of this preservation?
Search strategy	
Population	#1—edentulous patient
Intervention	#2—implant OR overdenture OR fixed bridge OR transmandibular implant OR full rehabilitation
Outcome	#3—bone density OR volume change OR bone formation OR bone apposition OR bone deposition OR bone preserving OR bone preservation OR bone maintain OR bone increase
Search combination	#1 AND (#2 OR #3)

#### Inclusion and exclusion criteria of studies

Inclusion criteria for the selected publications included the full-text articles written in English. Case reports also were included. Articles reported bone preservation or bone apposition even in clinical notifications or in the context were included. Studies that revealed improving bone density around implant were involved. The exclusion criteria included papers with only abstract available, while articles that deal only with discussing resorption through the remodeling process around cervical and/or implant body were excluded. Any articles related to abnormal conditions as maxillofacial patients, or treatments of systematic diseased patients, were not considered. Articles related to the effect of the implant on the bone for the opposing arch were excluded. Selection of articles is based on the title and the abstract reading. Some articles were excluded after full reading because of the absence of interest. Other articles were added from the existed citation bibliography (Table 1).

#### Data extraction and assessment

From the final included articles (*n* = 18), the following characteristics were tabulated:

- Author and the year of publishing
- Number of patients and implants mounted
- Average age of patients (exact age in case reports)
- Area of implant placement
- Type of prosthetic restoration
- Follow-up period
- Bony changes and declarations of the quality and quantity of remodeling (if present)
- Study design

### Results

**Fig. 1:**
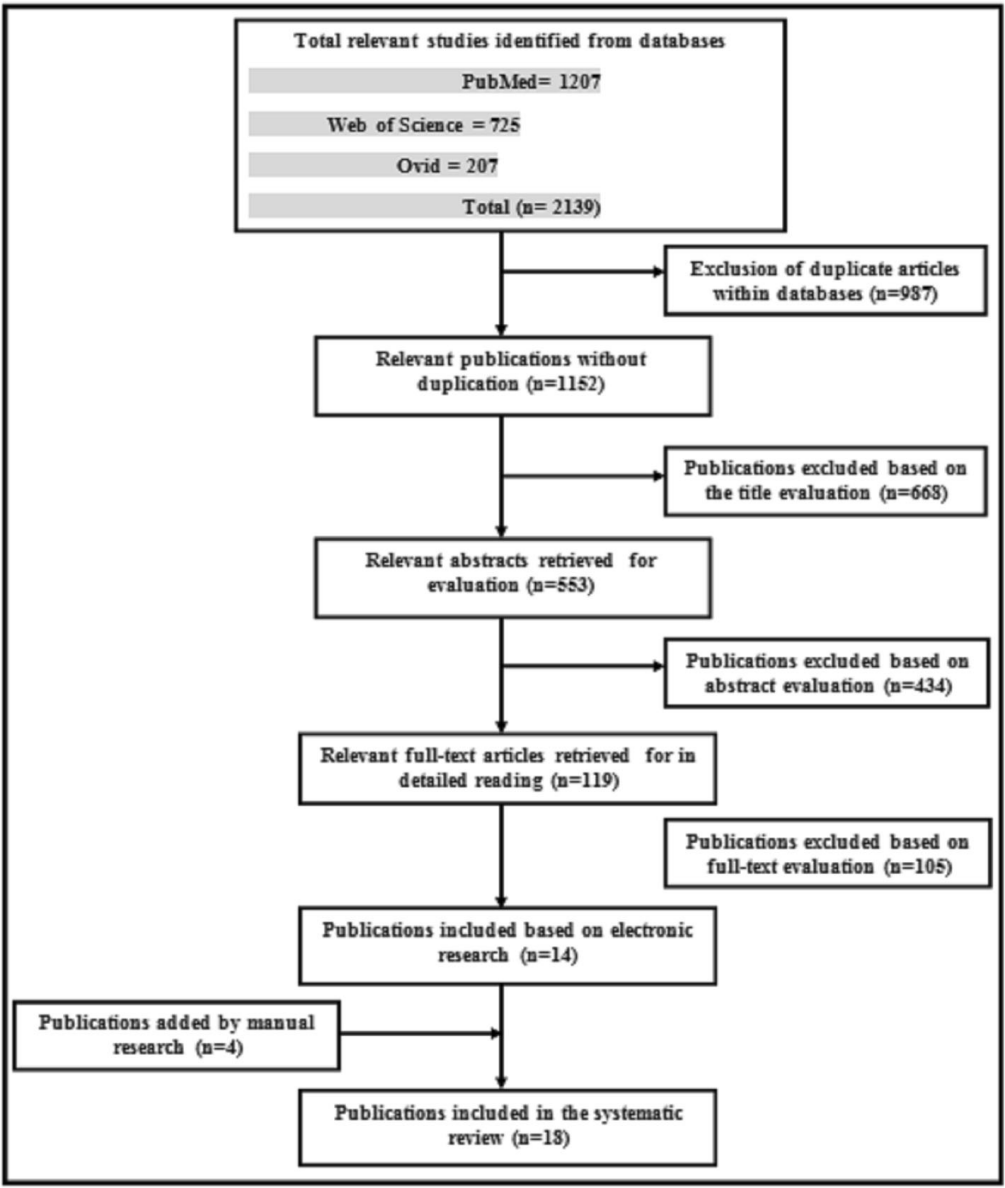
The final articles (*n* = 18) were selected according to the previous exclusion and inclusion criteria

Initial search retrieved a total 2139 citations. After discarding the repeated articles among databases, revising of titles extruded 668 articles as out of interest for this review. The remaining articles are disclosed more by abstract. We excluded 434 citations due to concentration on bone remodeling with an abnormal situation like compromised patients or with localized problems as maxillofacial patients or not focus on the process of ridge progression under different restorations. The rest of the articles were read carefully to extract conclusions or notices related to positive bone remodeling, bone preserving, bone formation, or increasing bone density of the alveolar ridge with implant. Articles which could not be retrieved as the reference numbers 21 and 22 in the article of Davis et al. [17] were not included. Other articles [18–21] (*n* = 4) were added manually after reading the bibliography of previous citations. The final articles (*n* = 18) were selected according to the previous exclusion and inclusion criteria (Fig. 1). All citations (*n* = 18) share the point of bone preservation with implant restoration or enhancement of bone density or, at least, reduction of alveolar bone resorption rate after implant placement (Table 2).

**Table 2:** Clinical studies included

	Patients		Intervention			Follow-up	Outcome		Study
Reference	No.	Age^a^	No. of implants	Position	Supra-structure		Change	Declarations	
(DAVIS et al. 1999) [17]	44	61.2	NG	Symphyseal	Fixed	6.6 Y^a^	VBH (−.8 to +3.3 mm)	–	R
(Powers et al. 1994) [32]	146	52	NG	TMI	Fixed	18–51 M	BF (+2 to 9 mm)	–	R
(Adell et al. 1981) [58]	410	53	2768	NG	Removable bridges	5–9 Y	BR (+)	–	R
(Mericske-Stern et al. 2002) [35]	41	61.2	4–6	Maxillary	Removable overdentures	4.1 Y^a^	BD (+)	Associated with radiographically visible decrease of the crestal bone.	P
(Kwakman et al. 1997) [31]	36	60	NG	TMI	Denture with cantilever extension	2.3 Y^a^	BF (+)	–	R
(Sennerby et al. 1988) [72]	41	51.3	NG	Symphyseal	Oerdenture	7.1 Y^a^	BR (−)	Implant bony area vs CD	R
(Friberg et al. 2000) [20]	49	63	247	NG	Fixed prosthesis	8 Y^a^	BF (+)	At two most distal implants	R
(Adell et al. 1986) [19]	16	53	95	Maxillomandibular	Removable bridge	3 Y^a^	BR (+)	Indicating a successive load-related remodeling	P
(Kordatzis et al. 2003) [74]	150	NG	300	Symphyseal	Bar overdenture	5 Y^a^	BR (−1 mm)^a^	Less bone atrophy with OD vs CD.	R
(Woven & Gotfredsen 1998) [78]	22	65	NG	Symphyseal	Overdentures	5 Y^a^	BF (+) BR (−)	Function related BF vs physiologic age-related BR	P
(Wright et al. 2002) [73]	44	53 Y, for overdenture and 64 Y for the fixed prosthesis	NG	NG	Overdentures and fixed prostheses	7.5 Y^a^	BR (−0.5 mm)^a^ BF (+0.5 mm)^a^	OD and FD, respectively	R
(Shaarawy & Aboelross 2013) [92]	14	58	14 + 28	Symphyseal and 2 in the first molar area	Overdenture	1 Y	BD (+)	Incisal reduction of BD followed by gradual increase in BD	P
(Taylor 1989) [21]	1	50 Y (not a mean)	5	NG	Fixed	32 M	VBH (3 mm)	–	CR
(Oikarinen & Siirila 1992) [60]	1	41 Y	6	NG	Fixed	8 Y^a^	VBH (+)	Nearly the doubled new BF	CR
(Betts et al. 1993) [18]	19	NG	NG	TMI	Fixed	53 M	VBH (+1.8 mm)^a^	In the saddle area and the most distal screw	R
(Dhima et al. 2013) [33]	81	NG	NG	NG	Fixed	9^a^	BF (+0.94 mm)^a^	–	R
(Mosnegutu et al. 2015) [81]	82	NG	NG	NG	Overdenture	10.5 Y^a^	BR (±)	No relevant posterior bone atrophy after loading	P
(Cooper et al. 2008) [79]	59	NG	118	Parasymphyseal	Overdenture	60 M	BF (+)	–	P

^a^Average

*NG* not given, *TMI* transmandibular implant, *Y* year, *M* month, *BF* bone formation, *VBH* vertical bone height, *BR* bone radiopacity, *BD* bone density, *BR* bone resorption, *CD* complete denture, *OD* overdenture, *FD* fixed denture, *R* retrospective, *P* prospective, *CR* case report, (*+*) increase, (*−*) decrease, (*±*) neutral

### Discussion

#### Implant role to enhance bone density

Apparently, there is an enduring adaptive process surrounding the implant which sustains the rigid interface between alveolar bone and implant after non-destructive surgical and loading procedures. Like other body bones, and according to Wolff’s law, bone has the ability to differentiate with different stresses applied [22]. This reform is started from the time of surgical conduction of implant and continued to support the implant to withstand the uploading forces [8, 9, 23–25]. Greater bone modification may occur at the alveolar bone around implants in partially edentulous patients [26, 27]. Roberts et al. [28] described the situation and the process as the ankylosed tooth which acts without bracing the attachment to bone and carries a heavy load. The adaptive modeling of endosseous implants, as a response to load, is a massive build-up of immature buttress-like skeleton which then decrease externally as the interior layers become more mature [28].

Like ankylosed tooth, implant-supported fixed prosthetic treatment might have a preservative effect on residual alveolar bone [29]. A radiographic-based quantitative study carried by Ichikawa et al. [30] displayed an improvement of bone density and bone formation related to load applied after a short period of implant service. With transmandibular implant, bone maintaining [18] or even formation and the highest increase in the bone were recorded [31]. The study for 146 patients with severely resorbed ridge treated with transmandibular implant denoted bone formation with all cases during follow-up period extended 51 months [32]. Within 9-year follow-up for 81 patients, Dhima et al. [33] denoted about 0.94 mm growth of bone. In a different study, peri-implant bone density grew around implants after 5 years of follow-up [34]. Even with questionable histological bony condition, as in the maxilla with sinus lifting, favorable bone density is noticed [35, 36]. Follow-up for more than 3 years for 44 installed fixtures revealed a consistent bone formation and elevation of lining sinus mucosa without bone graft. [37] This goes with the conclusion of Lundgren et al. [38] as the replaceable bone window allows bone formation with the implant after sinus lift.

Bone changes were reported after implant placement in three phases: healing, remodeling, and equilibrium. The remodeling phase is launched confronting the altered pattern of force transmission to the bone tissue. To withstand the applied functional load, continuous remodeling is conducted to reach a “steady state.” Mechanical stimulus is the primary bone modifier influenced by other in situ variables as hormonal, metabolic, genetic factors [39].

Clinically, as affordable as the strain, bone regeneration is configured, whereas the over-stimulation leads to adverse effects [40]. The bone around unloaded implants showed a low mineral density index. [41] The process of inducing more dense bone depends mainly on the loading protocol conducted [42]. The peri-implant bone around progressively loaded implants illustrated minimal crestal bone loss than the bone around implants placed conventionally, and the later cited extended increase in peri-implant bone density by time [10]. Based on radiological assessment, Barone et al. [43] stated statistical significant dense bone around immediate rather than unloaded oral implants.

Histochemically, the mechanical effect controls bone formation and mass modification with a percentage about 40% comparing to other growth-related factors as hormonal or cytokine deliver about 10% of the postnatal changes in bone strength and mass. Thereby, mineralization and tissue reformation by osteoblasts are co-related to the local mechanical environment [44]. The blood supply and nutrition are mandatory as osteoblast acting on the osteoid bone formation [45]. Bone tissue that experienced physiologic load is liable for osteogenic deformation [46]. Thus, strain must be in the physiologic bone limit (500–3000 μ strain) according to the elasticity modulus, while overstrain (>5000 μ strain) precipitates fibrogenesis [44, 47].

#### Variations of bone resorption and preserving with different restorations

Considering physiologic changes, the annual alveolar bone resorption is approximately fourfold more in mandible comparing to the maxilla [48]. A longitudinal monitoring of edentulous complete denture wearers admitted continuous reduction of the residual alveolar ridge throughout the study. The anterior part of the mandible showed the higher average of reduction compared to the estimated rate of the maxilla [49]. The variation between the jaws in alveolar bone reduction increased gradually during the first years of denture wearing. This evidenced the unfavorable response of the mandible to various functional stresses transmitted through the denture to the limited and diminished bearing area of the mandibular alveolar bone comparing to the maxilla [50].

Many procedures are used to recover denture foundation, but the majority is considered sophisticated techniques [51–53]. Observations tried to notify bone modifications with different types of implant-assisted restoration [23, 40, 54]. The clinical and radiographic investigations, detection of the altered mineral levels, or bone density within the bone may give a valuable data for the bony state around loaded implants [55–57]. In a prospective study, Adell et al. [19] noticed a reduction of probing depth around implants, resembling approximately that surround natural dentition which indicates active positive bone repair. A further sign of bone preserving is the radiopacity close to the fixture due to increasing in density [58]. Such radiopacity affirmed to increased bone volume and/or increased mineral content. Maxillary implants reflected more bone density rather than mandibular and distal cantilevered implants due to the stresses which may produce more unfavorable bone restoring condition. After 10 years of implant placement, a significant increase in peri-implant bone density was noted in a clinical study for 18 patients [59]. In two separate clinical reports, Taylor [21] reported patient’s complain with cantilevered part after 32 months of loading. He elaborated that with mandibular growing for about 3 mm. In the other case report by Oikarinen and Silrila [60], they mentioned new boney layer formation. Naert et al. [61] agreed with the role of the implant in residual ridge preservation even if there is no bone formation recorded.

#### Destructive and preservative role of implant overdenture

Occlusal load and different forces induced on the implant overdenture restoration, with the diminished supportive area, might be the main predisposing factor for bone resorption [62]. According to finite element analysis study, the available bearing area in case of complete denture is 4608.7 mm^2^ comparing to 2833.4 mm^2^ for the implant overdenture posteriorly which leads to an even pressure at the usage of complete denture comparing to higher load concentration on the posterior area available with overdenture [63]. This agreed with other study comparing hydrostatic pressure under the conventional versus implant overdenture which conceded the evenness of load distribution over the wide area of residual ridge, approximately 1926 mm^2^, and the volume average hydrostatic pressure at 10.670.8 kPa, in case of complete denture. While the tissue-bearing area reduced to 71% with two implants and to 60.5% for four implant-assisted overdenture, the corresponding hydrostatic pressure was 14.370.9 and 13.370.9 kPa, respectively. The peak of posterior stresses was recorded with the two implant-assisted overdentures [64]. In a clinical retrospective study for 10 years, there was a significant difference in posterior ridge resorption with overdenture assisted with two and/or four implants. This was interpreted as the improved oral function and increased bite force may lead to more force concentration which does not exist in floated conventional denture [65]. Due to the anchorage of the denture anteriorly in the symphyseal area, the axial direction of force and the free movement posteriorly may exert more resorption in comparing to preserved bone close to implant anteriorly [66, 67]. On the other hand, the best selection for supra-structure attachment with implant overdenture and the pre-intervention planning may reduce implant/ridge load by distributing forces in an even manner to act as the norm of implant-supported fixed dentures [68, 69]. Additional investigation elected the symphyseal implant overdenture as a good treatment modality without overestimation for posterior bone loss [70].

Despite age-related [71], local and/or systemic factors causing prolonged ridge resorption [9], authors reported the probability of preservative effect and overhaul to maintain the residual alveolar ridge with different restoration [70, 71]. In the previous study, bone formation was noticed with the distal implant in severely resorbed atrophied mandibular ridge [20]. Sennerby et al. [72] concluded that the treatment with tissue-integrated prostheses seems to reduce bone resorption in the mandible, probably owing to adequate favorable load to stimulate bone preservation. Patients rehabilitated with implant-stabilized mandibular overdenture demonstrated the preservation of posterior mandibular residual ridge from resorption by annual range +0.009 to − 0.048 mm, while patients with mandibular implant fixed cantilever prostheses elaborated bone apposition, in the same area, with 1.6% annually [73]. Additionally, Kordatzis et al. [74] concluded 1-mm annual reduction in bone loss at using implant overdenture comparing to the conventional denture. Davis et al. [17] noticed the liability of the severely resorbed mandible for regeneration. After more than 4 years of function, anterior implant regenerated bone in the mental foramen area created a mandibular canal that previously was unseen. Even with loaded comparing to non-loaded implant in the same patient, the loaded implant demonstrated more bone preservation [75]. Within the 4-year study of implant-supported overdenture, preservation and gaining of more bones were preserved. Clinical examination revealed 0.8 mm mean annual marginal bone loss during the first year and 0.1 mm in the following years [76]. Also, the bony area close to the implants has advantaged reduction in bone resorption. The reduced resorption rate with implant-supported overdenture is significantly proportional to the distance from the distal implant which contributes to protecting the posterior residual ridge from excessive loading [74, 77]. In a retrospective 5-year study, 22 patients with bar retained and freestanding implant overdenture patients demonstrated a significant preservation of bone surrounding implant. The increased function after prosthetic rehabilitation reflected load-related bone deposition which minimized the physiologic age-related mandibular bone mineral content loss regardless the attachment system [78]. A non-significant bone gain was recorded with 59 patients after wearing overdenture for 60 months [79]. Another clinical investigation, extended for 8 years, proved the usage of bar-assisted overdenture in the treatment of severely resorbed alveolar ridge represented preservation and minimal rate bone resorption regardless the design of bar [80]. The same conclusion was announced by Mosnegutu et al. [81] after 10 years of follow-up for some cases. Transmission of load axially toward implant followed by posterior load on the ridge initiated a negative consequence on the posterior bone and preservative positive alveolar bone response around osseointegrated implants [82]. Development of high strain in the alveolar region is inevitable causing crestal bone resorption [83, 84]. Strain levels in peri-implant bone are reduced as the insertion depth of the implant increased [85]. The chance of bone preserving is high in normal range of load and in the absence of abnormal overload conditions [69].

As most of the previous studies declared the favorable bone preservation of the residual alveolar ridge anteriorly around implants, biomechanically, and according to finite element analysis, bone modifying shows variations depending on the cancellous or cortical nature. Bone density is enhanced gradually from the third month to the end of the first year of loading coming stable after 30 months. Whoever, bone adaptive activities expose more impact on cancellous areas more than other parts [86]. Favorable bone remodeling may occur close to implants in partially edentulous patient because of the chance to be surrounded by alveolar rather than basal bone [26]. Thus, the cortical bone reveals more force concentration and liability to resorb rather than the cancellous bone showing more liability to accommodate with the induced forces [87, 88]. According to Chou et al. [89], the topography of the fixture reported alternative responses to load promotes a variation of bone remodeling. Due to the diminished bone surrounding, mini-implants revealed less bone adaptive capacity [90]. The threaded fixture represented bone apposition at the tip of threads and subsequent resorption at the bottoms. The non-threaded smooth implant revealed increasing in bone density at the apical section with a connection of high-density region to the cortical bone. According to Li et al. [91], positive density was illustrated deeper to the implant surface which may be due to the mechanical stimulus on the favorable cancellous bone.

## Conclusions

Within the limitation of this review and based on previous studies, implant restoration has a noticeable residual alveolar ridge preservation which varies from reducing rate of physiologic resorption to bone apposition. However, the extension of this preservation from the implant to surrounding bony area, horizontally and vertically, is unknown. So, further studies are needed to elaborate the extension of preservation and the influencing factors.
